# Examining Measurement Invariance in the Assessment of Language Across Literacy Status in India and Mexico

**DOI:** 10.1093/geroni/igaf122.2130

**Published:** 2025-12-31

**Authors:** Alexa Gonzalez, Gelan Ying, Pranali Khobragade, Miguel Arce Rentería, Zachary Kunicki, Emily Briceño

**Affiliations:** University of Houston, Houston, Texas, United States; University of Florida, Gainesville, Florida, United States; University of Southern California, Los Angeles, California, United States; Columbia University Irving Medical Center, New York, New York, United States; Warren Alpert Medical of Brown University, Providence, Rhode Island, United States; University of Michigan Medical School, Ann Arbor, Michigan, United States

## Abstract

Accurate neuropsychological assessment is essential for evaluating cognitive decline, particularly across culturally and linguistically diverse populations with varying levels of formal education. Ensuring cognitive tests measure the same constructs across those with varying levels of literacy is crucial, especially when assessing language abilities. Therefore, we evaluated the measurement invariance properties of the language domain across literate and illiterate older adults in India and Mexico. Data were drawn from the Longitudinal Aging Study in India– Diagnostic Assessment of Dementia’s (LASI-DAD) and the Mexican Health and Aging Study Cognitive Aging Ancillary Study’s (Mex-Cog) Harmonized Cognitive Assessment Protocol (HCAP). In each cohort, a multiple-group confirmatory factor analysis was used to determine the level of invariance achieved in the latent language factor, composed of items measuring verbal fluency, item naming, and comprehension. As demonstrated by acceptable fit, results supported configural invariance in LASI-DAD (CFI=.94; RMSEA=.03; SRMR=.03) and Mex-Cog (CFI=.93; RMSEA=.04; SRMR=.03). However, relative fit statistics significantly differed between configural and metric invariance in LASI-DAD (Δχ²=49.88; Δdf = 13; p < .001) and Mex-Cog (Δχ²=39.62; Δdf = 9; p < .001); therefore, full metric invariance is not supported, suggesting language measurement differs by literacy status. This study highlights the need for careful evaluation of measurement properties when assessing language function across culturally and linguistically diverse adults with varied levels of literacy. Future studies leveraging these HCAP cohorts should adjust the language domain due to these measurement differences driven by literacy and evaluate whether other cognitive domains are similarly affected.

